# miR-34a Repression in Proneural Malignant Gliomas Upregulates Expression of Its Target PDGFRA and Promotes Tumorigenesis

**DOI:** 10.1371/journal.pone.0033844

**Published:** 2012-03-27

**Authors:** Joachim Silber, Anders Jacobsen, Tatsuya Ozawa, Girish Harinath, Alicia Pedraza, Chris Sander, Eric C. Holland, Jason T. Huse

**Affiliations:** 1 Department of Pathology, Memorial Sloan-Kettering Cancer Center, New York, New York, United States of America; 2 Human Oncology and Pathogenesis Program, Memorial Sloan-Kettering Cancer Center, New York, New York, United States of America; 3 Computational Biology Center, Memorial Sloan-Kettering Cancer Center, New York, New York, United States of America; 4 Department of Cancer Biology and Genetics, Memorial Sloan-Kettering Cancer Center, New York, New York, United States of America; University de Minho, Portugal

## Abstract

Glioblastoma (GBM) and other malignant gliomas are aggressive primary neoplasms of the brain that exhibit notable refractivity to standard treatment regimens. Recent large-scale molecular profiling has revealed distinct disease subclasses within malignant gliomas whose defining genomic features highlight dysregulated molecular networks as potential targets for therapeutic development. The “proneural” designation represents the largest and most heterogeneous of these subclasses, and includes both a large fraction of GBMs along with most of their lower-grade astrocytic and oligodendroglial counterparts. The pathogenesis of proneural gliomas has been repeatedly associated with dysregulated PDGF signaling. Nevertheless, genomic amplification or activating mutations involving the PDGF receptor (PDGFRA) characterize only a subset of proneural GBMs, while the mechanisms driving dysregulated PDGF signaling and downstream oncogenic networks in remaining tumors are unclear. MicroRNAs (miRNAs) are a class of small, noncoding RNAs that regulate gene expression by binding loosely complimentary sequences in target mRNAs. The role of miRNA biology in numerous cancer variants is well established. In an analysis of miRNA involvement in the phenotypic expression and regulation of oncogenic PDGF signaling, we found that miR-34a is downregulated by PDGF pathway activation *in vitro*. Similarly, analysis of data from the Cancer Genome Atlas (TCGA) revealed that miR-34a expression is significantly lower in proneural gliomas compared to other tumor subtypes. Using primary GBM cells maintained under neurosphere conditions, we then demonstrated that miR-34a specifically affects growth of proneural glioma cells *in vitro* and *in vivo*. Further bioinformatic analysis identified PDGFRA as a direct target of miR-34a and this interaction was experimentally validated. Finally, we found that PDGF-driven miR-34a repression is unlikely to operate solely through a p53-dependent mechanism. Taken together, our data support the existence of reciprocal negative feedback regulation involving miR-34 and PDGFRA expression in proneural gliomas and, as such, identify a subtype specific therapeutic potential for miR-34a.

## Introduction

Malignant gliomas—particularly glioblastoma (GBM), the most common and aggressive adult variant—continue to cause a disproportionate degree of morbidity and mortality within human oncology and remain soberingly refractive to conventional therapeutic options [1]. Recent integrated genomics has convincingly demonstrated biologically distinct subclasses within malignant glioma that transcend conventional histopathological boundaries, with each characterized by differing patterns of driving molecular abnormalities [2], [3], [4]. Perhaps the broadest of these subclasses has been termed “proneural” and includes both a large fraction of GBMs along with most of their lower-grade astrocytic and oligodendroglial counterparts. The pathogenesis of proneural gliomas has been strongly linked to dysregulated PDGF signaling and the activation of downstream oncogenic signaling networks [4]. And while amplification or activating mutations involving the PDGF receptor gene (*PDGFRA*) characterize a significant subset of proneural GBMs [3], [4], alternative non-genomic mechanisms driving PDGF signaling, particularly in lower-grade tumors, remain unclear. In such cases, the involvement of epigenomic and/or pretranslational regulatory systems seems likely.

miRNAs are short (∼22 nucleotide) single stranded RNAs that repress gene expression by binding loosely complementary sequences in the 3′-untranslated regions (3′-UTRs) of target mRNAs [5]. Each miRNA likely interacts with numerous mRNA transcripts, a promiscuity that speaks to the potential of individual miRNAs to serve as “master regulators” mediating complex biological phenotypes [6]. Nevertheless, the relative importance of specific miRNA/mRNA interactions can depend heavily on physiological and cellular context, underscoring the absolute necessity of tissue and disease-specific experimental validation. Many research groups, including our own, have directly implicated a number of specific miRNAs in the pathogenesis of malignant glioma, both as tumor suppressors and oncogenes [7]. Furthermore, miRNA signatures have recently been demonstrated to stratify GBMs into defined biological subclasses, with each demonstrating intriguing links to distinct cellular lineages within the central nervous system [8].

We sought to identify and characterize biologically relevant miRNA-mediated regulatory networks involved in proneural gliomagenesis. In this report, we use a combination of experimental and computational methodologies to demonstrate that miR-34a is downregulated by oncogenic PDGF signaling, and that its expression level is negatively correlated with proneural subclass in GBM. We then show that miR-34a specifically inhibits growth in proneural glioma cells both *in vitro* and *in vivo* and identify PDGFRA as a direct and functionally consequential miR-34a target. Finally, we demonstrate that the regulation of miR-34a expression by PDGF signaling likely operates through a p53-independent mechanism. Our findings thus identify a reciprocal negative feedback loop influencing oncogenic receptor tyrosine kinase (RTK) signaling whose fundamental dysregulation in proneural gliomas contributes to tumorigenesis.

## Results

### miR-34a is downregulated in proneural GBMs and in response to activated PDGF signaling

**Figure 1:**
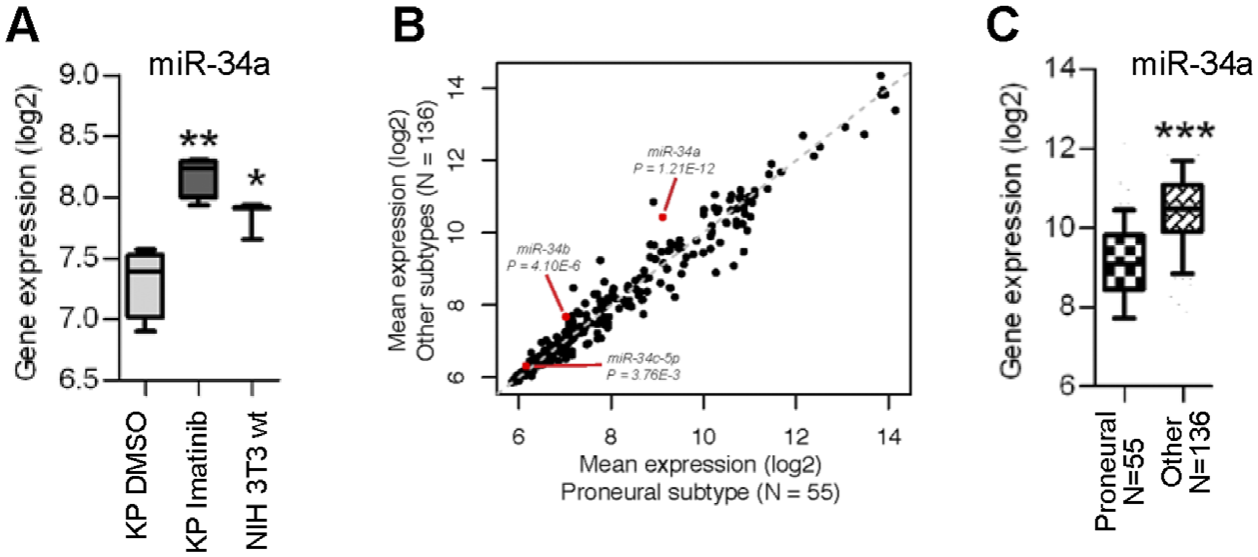
miR-34a is selectively downregulated in proneural glioma, likely in response to activated PDGF signaling. **A**) RNA was extracted from KP cells treated with DMSO control, KP cells treated with imatinib for 72 hours, and parental NIH 3T3 cells treated with DMSO control. miRNA expression profiling identified miR-34a as being responsive to PDGF signaling. **B**) Data from TCGA demonstrate a strong negative correlation of miR-34a with proneural subclass. **C**) Expression of miR-34a in proneural gliomas and in gliomas of other subtypes. *, P<0.05. **, P<0.005. ***, P = 1e-12.

Given the central role likely played by dysregulated PDGF signaling in proneural gliomagenesis, we wanted to identify miRNAs whose expression levels responded to changes in pathway activation status. To do this, we utilized an NIH-3T3 cell line harboring a unique fusion protein (KP) composed of the extracellular domain of KDR (VEGF receptor II) and the intracellular domain of PDGFRA [9]. KP overexpression morphologically and functionally transforms NIH-3T3 cells in a manner that is completely reversible by pharmacological inhibition with imatinib. Profiling of KP-expressing NIH-3T3 cells revealed a number of miRNAs whose expression levels varied in response to oncogenic PDGF signaling. Among the most significantly downregulated miRNA species was miR-34a, whose repression was completely reversible with imatinib treatment (FIG. 1A). Additionally, we found that neither of the miR-34a homologues miR-34b and -34c exhibited statistically significant expression changes in this experimental paradigm (data not shown). These findings indicated that dysregulated PDGF signaling selectively represses miR-34a. To determine whether miR-34a is also specifically downregulated in proneural GBMs we analyzed publically available miRNA expression data from 191 primary GBMs profiled by the Cancer Genome Atlas (TCGA). We found that miR-34a levels exhibited a strong negative correlation with proneural subclass (*P* = 1.2×10^−12^; FIG. 1B–1C). Once again, miR-34b and -34c failed to exhibit similarly robust associations (FIG. 1B). Coupled with our KP cell line data, these findings indicate that miR-34a is selectively downregulated in proneural GBMs, likely in response to oncogenic PDGF signaling.

### miR-34a specifically inhibits proliferation and tumorigenesis in proneural glioma cells

**Figure 2:**
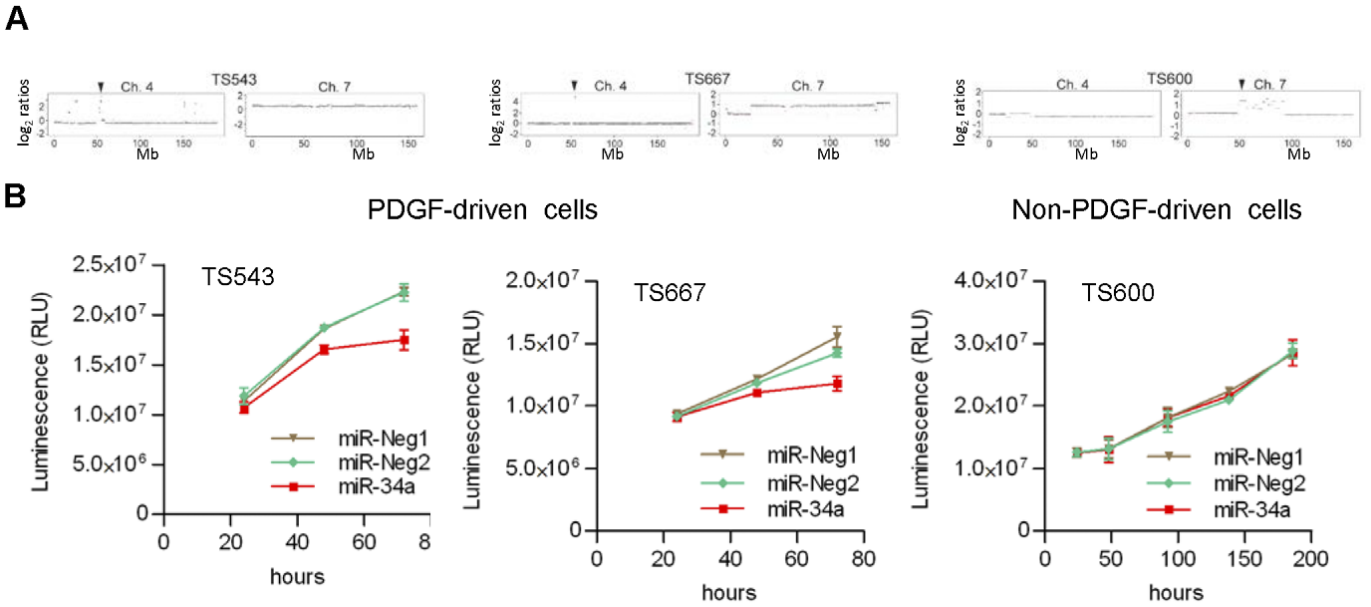
miR-34a specifically inhibits proliferation of proneural glioma cells. **A**) aCGH analyses of cell lines used. Proneural TS543 and TS667 cells both harbor PDGFRA amplification on chromosome 4, and mesenchymal TS600 cells harbor EGFR amplification on chromosome 7. **B**) Cells were transiently transfected with miR-34a mimics or control oligonucleotides and for assayed cell numbers over 3–8 days by MTT assay.

To determine the functional importance of miR-34a downregulation in proneural malignant gliomas, we restored its expression in three human glioma cell lines cultured as neurospheres in stem-like conditions (TS543, TS667, and TS600). Prior array-comparative genomic hybridization (aCGH) had demonstrated focal *PDGRA* amplification (chromosome 4q) in both TS543 and TS667 cell lines (FIG. 2A), and each demonstrated strong proneural character as determined by validated transcriptional analysis (Huse, J.T., Kastenhuber, E.R, and Brennan, C.W., unpublished work). By contrast, TS600 was characterized by *EGFR* amplification (chromosome 7p) by aCGH (FIG. 2A) and mesenchymal subclass by expression analysis. Exogenously driven miR-34a expression significantly slowed proliferation in both TS543 and TS667 cells, but not TS600 cells as assessed by MTT assay (FIG. 2B). Furthermore, robust miR-34a overexpression in these experiments was validated in both responsive (TS543) and unresponsive (TS600) cell lines (FIG. S1). These findings reveal a selective inhibitory capability of miR-34a on cell proliferation that is largely restricted to proneural subclass.

**Figure 3:**
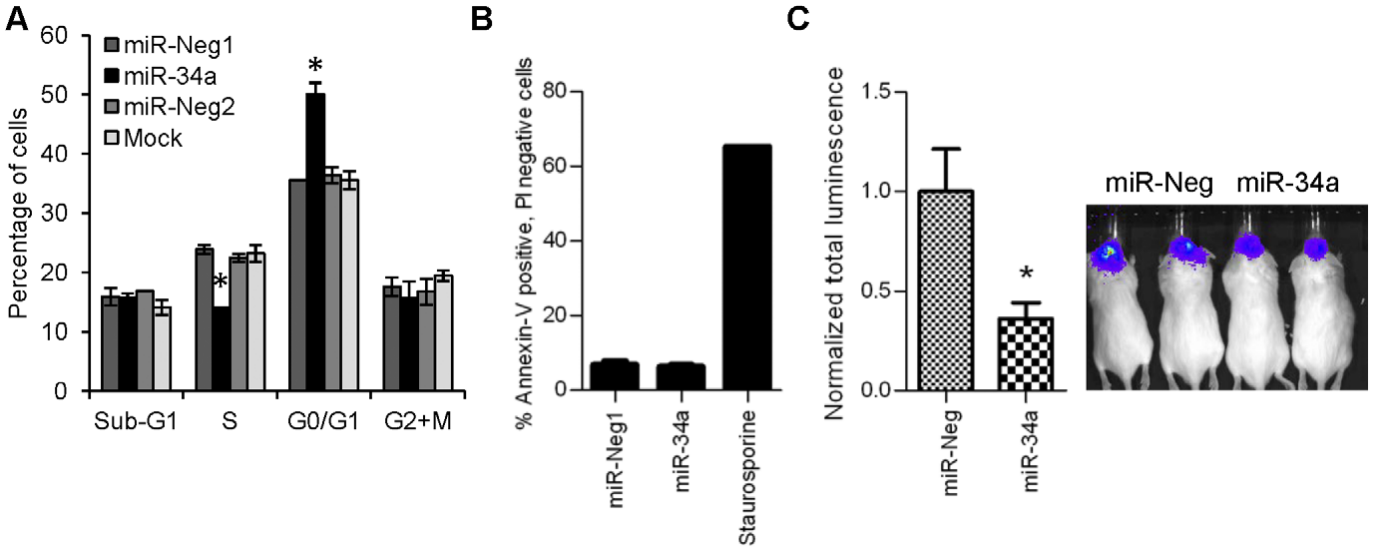
miR-34a induces cell cycle arrest and inhibits tumorigenesis in proneural GBM cells. **A**) 48 hours post-transfection, proneural TS543 were pulsed with BrdU and assayed for cell cycle distribution and **B**) apoptosis by Annexin V flow cytometry. Staurosporine was added as positive control. **C**) *Ex vivo*-transfected TS453 cells stably expressing a luciferase reporter plasmid were xenografted into the brains of ICR scid mice and tumor formation and growth was followed over 11 days. Error bars represent standard deviation within a single experimental set containing multiple replicates. Similar results were obtained in at least 3 independent experiments. *, P<0.05. **, P<0.005.

To more precisely determine the biological impact of miR-34a on proneural gliomagenesis, we performed cell cycle distribution analysis on TS543 cells pulsed with BrdU. We found that miR-34a transfection markedly decreased the number of cells in S-phase by 39% (*P*<0.05) with a concomitant increase in the number of cells in G_0_/G_1_, relative to both control oligonucleotide and mock transfection (FIG. 3A). We also evaluated the effect of miR-34a on apoptosis induction but did not observe any increase in annexin V staining in miR-34a transfected TS543 cells (FIG. 3B). Finally, to probe the functional relevance of miR-34a to proneural gliomagenesis *in vivo*, we xenografted either miR-34a or control oligonucleotide transfected TS543 cells into the brains of ICR scid mice. A stably expressed luciferase construct allowed for monitoring of tumor growth by luminescence. This analysis revealed a significant inhibitory effect of miR-34a on tumor formation (64% reduction in tumor size, *P*<0.05) over the course of 11 days (FIG. 3C). Taken together, our data indicate that miR-34a represses proneural gliomagenesis both *in vitro* and *in vivo*, primarily by interfering with cell cycle progression.

### miR-34a directly targets PDGFRA transcript to facilitate proneural gliomagenesis

**Figure 4:**
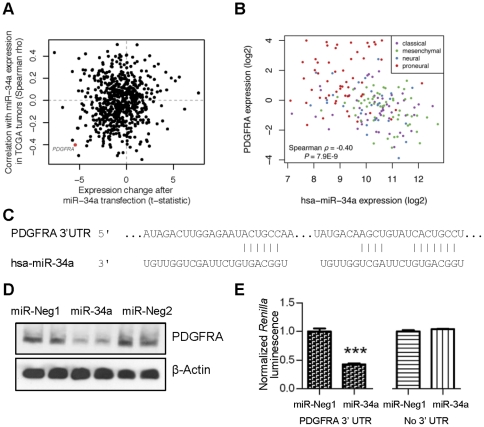
miR-34a targets PDGFRA. **A**) Scatter plot showing expression change (quantified by a moderated t-statistic) following miR-34a overexpression in TS543 cells and correlation with miR-34a expression in TCGA GBM tumors (Spearman correlation) for all miR-34a predicted mRNA targets. PDGFRA mRNA is highlighted in red. **B**) Integrated molecular profiling data from TCGA show that PDGFRA transcript exhibits a negative association with miR-34a. Expression subclasses are indicated (proneural-red, neural-blue, mesenchymal-green, and classical-purple). **C**) The two predicted miR-34a binding sites in the 3′ UTR of PDGFRA. **D**) TS543 cells were transfected with either miR-34a or control oligonucleotides for 24 hours, and PDGFRA protein levels were measured by immunoblotting. **E**) miR-34a was co-transfected with a PDGFRA 3′ UTR luciferase reporter construct into TS543 cells and assayed for luciferase 24 hours post-transfection. As a control, the parental luciferase reporter construct not containing the PDGFRA 3′ UTR segment was used in parallel studies. Error bars represent standard deviation within a single experimental set containing multiple replicates. Similar results were obtained in at least 3 independent experiments. ***, P<0.0001.

To identify potential targets of miR-34a, we again utilized integrated molecular profiling data from TCGA, this time selecting mRNAs predicted to interact with miR-34a whose expression was also anticorrelated with that of the miRNA. In parallel, we also performed transcriptional profiling on TS543 cells, focusing on mRNAs whose levels decreased in response to miR-34a transfection as compared to control oligonucleotide. Plotting these results orthogonally to each other revealed PDGFRA transcript as one of the most negatively correlated mRNA candidates by this combined analysis, consistent with a direct miRNA/mRNA regulatory interaction (FIG. 4A). Perhaps not surprisingly, when the reciprocal relationship of miR-34a and PDGFRA was examined for all TCGA tumors after expression subclass stratification, PDGFRA levels were highest in proneural GBMs (FIG. 4B).

Examination of the PDGFRA 3′ UTR revealed two potential evolutionary conserved binding sites for miR-34a (FIG. 4C). To determine whether miR-34a directly represses PDGFRA mRNA, we performed western blots on lysates from TS543 cells transfected with either miR-34a or control oligonucleotide and found a dramatic decrease in PDGFRA protein levels associated with miR-34a overexpression (FIG. 4D). To confirm that miR-34a directly complexes with PDGFRA transcript, we cloned the segment of its 3′ UTR harboring these two binding sites downstream of a luciferase reporter cassette and expressed the resulting plasmid in TS543 cells. Co-transfection with miR-34a resulted in a significant 57% decrease (*P*<0.0001) in luminescence relative to a control oligonucleotide (FIG. 4E). Moreover, an analogous experiment utilizing the parental luciferase reporter construct not containing the PDGFRA 3′ UTR segment failed to demonstrate reduced luminescence in response to miR-34a overexpression. These findings confirm miR-34a regulates PDGFRA transcript through direct interactions with its 3′ UTR.

**Figure 5:**
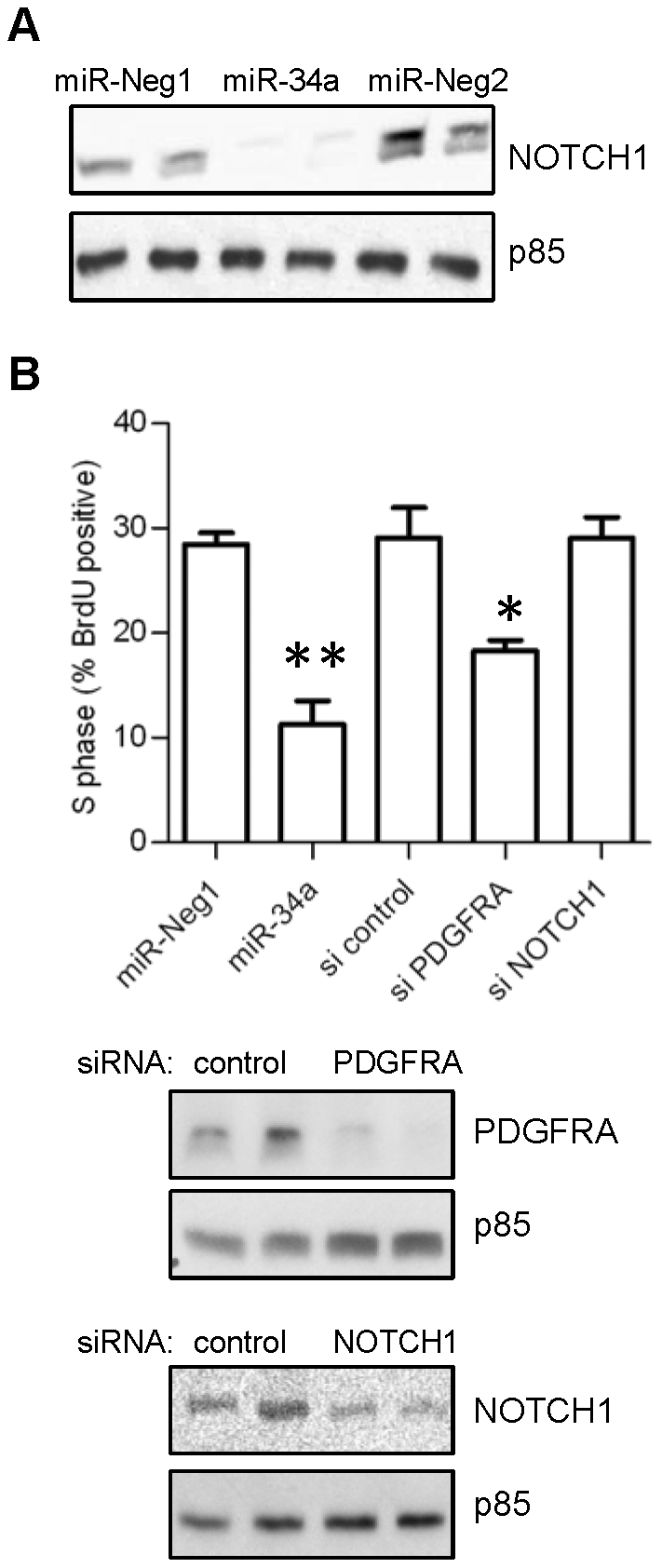
PDGFRA represents the most functionally consequential miR -34a target. **A**) TS543 cells were transfected with either miR-34a or control oligonucleotides for 24 hours, and NOTCH1 protein levels were measured by immunoblotting. **B**) 48 hours post-transfection, TS543 cells transfected with either miR-34a, siRNAs against PDGFRA, siRNAs against NOTCH1, or control oligonucleotides were pulsed with BrdU and assayed for cell proliferation by S-phase BrdU incorporation. siRNAs against PDGFRA and NOTCH1 are effective at reducing PDGFRA and NOTCH1 protein levels, respectively, as measured by immunoblotting 24 hours post-transfection. Error bars represent standard deviation within a single experimental set containing multiple replicates. Similar results were obtained in at least 3 independent experiments. *, P<0.05. **, P<0.005.

Several additional miR-34a targets have been reported thus far in a variety of cell and tissue types. Specifically in the context of malignant glioma, these include NOTCH1, NOTCH2, MET, and CDK6 [10]. To evaluate whether miR-34a-mediated regulation of these targets contributes to the miRNA's functional impact on proneural gliomagenesis, we performed western blots in miR-34a and control oligonucleotide transfected TS543 cells. We found that miR-34a overexpression only exhibited strong repressive effects on NOTCH1, with NOTCH2, MET, and CDK6 levels essentially remaining unchanged (FIG. 5A and S2). To determine the extent to which selective PDGFRA and NOTCH1 repression recapitulates to the biological effects of miR-34a in proneural glioma, we transfected TS543 cells with siRNAs specific for either mRNA target and monitored cell proliferation by S-phase BrdU incorporation (FIG. 5B). In this experimental context, only PDGFRA knockdown reduced BrdU uptake (37% reduction, *P*<0.05) comparably to miR-34a overexpression (60% reduction, *P*<0.005), with NOTCH1 knockdown having no observable effect. These findings demonstrate that PDGFRA repression by miR-34a promotes proneural gliomagenesis and, furthermore, suggest that the PDGFRA represents the most functionally consequential miR-34a target.

### miR-34a repression in proneural gliomas is only modestly dependent on p53

**Figure 6:**
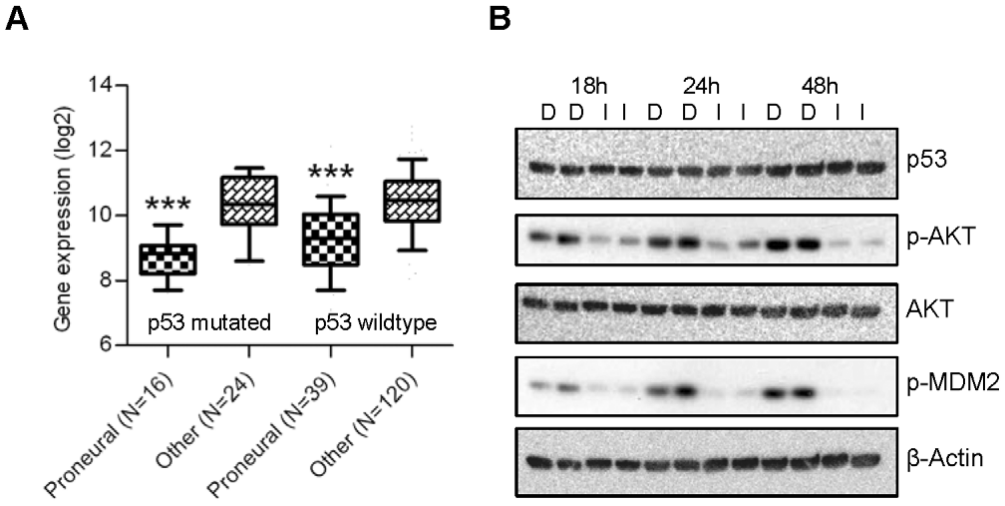
miR-34a repression in proneural GBM is likely p53-independent. **A**) Data from TCGA show that miR-34 expression is downregulated in proneural tumors in both p53 wild-type and p53 mutant subsets. **B**) KP cells were treated with imatinib (I) or DMSO (D) over 48 hours. Levels of p53, p-AKT, AKT, and p-MDM2 were determined by immunoblotting. ***, P<0.0001.

Several reports have shown that miR-34a is transcriptionally activated by p53 [11], [12], [13], [14]. To determine if miR-34a downregulation in proneural gliomas is p53-dependent, we once again applied both bioinformatic and experimental approaches. Integrating expression and genomic data from TCGA, we found significantly reduced miR-34a expression in proneural GBMs compared to those of other subclasses irrespective of p53 genomic status (*P*<0.0001; FIG. 6A). While miR-34a levels were somewhat lower in p53-mutant proneural tumors compared to their p53-wild type counterparts (*P* = 0.048), the impact of proneural molecular subclass on miR-34a expression clearly predominated. We also utilized the KP NIH-3T3 system to assess whether PDGF-induced miR-34a repression is dependent on p53. Activated PI3K/AKT signaling, a canonical effector pathway mobilized by RTKs like PDGFRA, has been shown to induce p53 degradation through phosphorylation of MDM2 [15]. In KP expressing cells, we found high-levels of both p-AKT and p-MDM2 by western blot that were each suppressed with imatinib, consistent with earlier reports. However, somewhat surprisingly, we were not able to demonstrate any change in p53 expression across multiple time points (FIG. 6B). These results, coupled with our bioinformatic data, indicate that p53 exerts, at best, only a modest effect on miR-34a expression in proneural gliomas.

## Discussion

The ineffectiveness of standard therapies in the treatment of malignant gliomas signals an urgent need for the development of disease-relevant targeted agents. This process itself requires a more complete understanding of underlying glioma biology, particularly with regard to its well-established variability at the molecular level. Several miRNA-mediated networks have been directly implicated in gliomagenesis, and recent work has shown that robust miRNA signatures designate distinct GBM subclasses, highlighting the relevance of miRNA biology to the analysis of molecular heterogeneity in malignant glioma [7], [8]. In the present study, we aimed to identify and characterize miRNAs involved in the pathogenesis of proneural gliomas using an approach that repeatedly combined *in vitro* methodologies with existing bioinformatic resources. As such, the ready availability of TCGA profiling data for GBM was an essential component of our basic strategy. Our findings indicate that proneural gliomas are specifically characterized by miR-34a downregulation with subsequent derepression of the miRNA's downstream target PDGFRA, a process that promotes tumorigenesis both *in vitro* and *in vivo*. The restriction of this regulatory interaction to proneural gliomas is perhaps not surprising given the highly distinctive molecular profiles exhibited by different GBM expression subclasses, and the central role of PDGFRA in proneural gliomagenesis. miRNA behavior and target profiles are known to be highly dependent on cellular context. Indeed, specific miRNAs have even been shown to behave as both oncogenes and tumor suppressors in different cancer variants [16].

miR-34a was originally identified as a likely tumor suppressor miRNA and a downstream transcriptional target of p53 [11], [12], [13], [14]. Prior reports have shown that miR-34a is downregulated in GBM compared to normal brain, and that it inhibits cell proliferation, survival, and invasion in adherent glioma cell lines by targeting MET, NOTCH1, NOTCH2, and CDK6 [10]. Additionally, miR-34a appears to promote differentiation in glioma cells grown in stem-like conditions [17]. Our findings indicate that PDGFRA represents a crucial target of miR-34a in the setting of proneural gliomagenesis. Our examination of other transcripts known to interact with miR-34a in GBM revealed that only NOTCH1 was significantly repressed by the miRNA in *bona fide* proneural glioma cells. Moreover, specific siRNA knockdown of NOTCH1 failed to demonstrate functional consequences, while PDGFRA knockdown yielded significantly reduced BrdU incorporation, emphasizing functional relevance. Nevertheless, we anticipate that other important miR-34a targets remain to be identified, particularly given that selective PDGFRA knockdown does not fully recapitulate the effects of miR-34a on cell proliferation in proneural glioma cells.

miR-34a repression in proneural gliomas appears to result directly from enhanced PDGF signaling. We found that constitutive activity in the PDGF pathway directly downregulates miR-34a expression in a manner that is completely reversible by imatinib administration. The well-established role of p53 in the transcriptional activation of miR-34a prompted us to investigate whether miR-34a repression in proneural gliomas is mediated through a p53-dependent mechanism. Indeed, earlier work has identified a potential conduit for such transcriptional regulation through p-AKT and p-MDM2 [15]. However, our western blot analysis in proneural TS543 cells failed to demonstrate significant changes in p53 levels in response to imatinib, despite robust modulation of both p-AKT and p-MDM2 levels. These finding indicate, somewhat surprisingly, that upstream regulation of miR-34a is, at best, only partially regulated by a p53-dependent mechanism and that alternative molecular pathways are likely involved in proneural GBMs. Consistent with this conjecture are TCGA data demonstrating that proneural subclass, rather than p53 genomic status, is most predictive of miR-34a expression levels. Additionally, the fact that miR-34b and -34c, which are also regulated by p53 [12], are not similarly repressed by dysregulated PDGF signaling provides further support for the contribution of p53-independent mechanisms.

In summary, we identify a miRNA-mediated network that promotes tumorigenesis in a specific glioma subtype by deregulating that subtype's single most defining oncogenic driver. Disrupting the homeostatic equilibrium between miR-34a and PDGFRA potentially gives rise to a cancer-promoting, feed-forward loop, one that could ultimately lead to both cell proliferation and tumor formation. Intriguingly, such a mechanism could conceivably drive gliomagenesis even in the absence of canonical genomic aberrations (e.g. mutations or copy number gains) in core signaling pathway components. This consideration is particularly relevant to proneural gliomas, which, despite their strong associations with dysregulated PDGF signaling, only harbor mutations and or amplifications of PDGFRA in a minority of cases. On a related note, these findings also serve to further emphasize the therapeutic potential of inhibiting PDGF signaling in the proneural subtype of malignant glioma.

## Materials and Methods

### Ethics statement

Primary GBM samples were obtained after approval by the Memorial Sloan-Kettering Cancer Center Institutional Review Board under the auspices of an existing blanket tissue collection protocol after written informed consent. This study was performed in strict accordance with the recommendations in the Guide for the Care and Use of Laboratory Animals of the National Institutes of Health. Animal experiments were conducted using protocols approved by the Institutional Animal Care and Use Committee of Memorial Sloan-Kettering Cancer Center (Protocol 09-09-017).

### Chemicals, miRNA- and siRNA-oligonucleotides

Imatinib was purchased from LC laboratories (Woburn, MA). Staurosporine was from Sigma-Aldrich (St. Louis, MO). miRIDIAN miRNA mimic negative controls #1 and #2, miRIDIAN miRNA mimics (hsa-miR-34a), PDGFRA and NOTCH1 targeting ON-TARGETplus SMARTpool siRNAs and control were purchased from Dharmacon (Lafayette, CO).

### Quantitative reverse transcriptase polymerase chain reaction

Total RNA was extracted using the miR-Vana RNA isolation system (Applied Biosystems, Foster City CA). Expression of miR-34a was measured using TaqMan® miRNA assays system (Applied Biosystems, Foster City, CA) according to the manufacture's instruction. RNU6B was used as endogenous control.

### Copy number assessment in glioma cell lines

Genomic DNA was extracted from primary tumors using standard techniques. DNA was then digested and labeled and hybridized to 244K CGH arrays according to manufacturer guidelines (Genomic DNA labeling kit PLUS, Agilent, Santa Clara, CA). Normal male genomic DNA (Promega, Madison, WI) was used as a reference. Array scanning, segmentation of raw data, and plotting was performed as previously described [4].

### Cell culture and transfections

NIH 3T3 cells and NIH3T3 cells expressing the KDR-PFGFRA (KP) fusion protein were obtained as previously described [9]. KP and control NIH 3T3 cells were cultured under 5% CO_2_ at 37°C in Dulbecco's Modified Eagle's medium (ATCC: 30-2002) with 10% heat-inactivated calf serum (Colorado Serum Co. Denver, CO). Tumor sphere (TS) cells were isolated from primary human glioblastomas by disassociation into single cell suspension using Accumax (Innovative Cell Technologies, San Diego, CA) as previously described. Cells were filtered through a 100 µm filter, and plated in NeuroCult NS-A proliferation media (Stem Cell Technologies, Vancouver, BC) supplemented with EGF (20 ng/ml; R&D Systems, Minneapolis, MN), FGF (10 ng/ml; Peprotech, Rocky Hill, NJ), and heparin (2 µg/ml, Stem Cell Technologies, Vancouver, BC). Oligonucleotides were transfected to a final concentration of 100 nM using the HiPerFect Transfection Reagent (Qiagen, Valencia, CA) according to the manufacturer's instructions. Imatinib/Gleevec treatment of KP NIH 3T3 cells were performed at 10 uM.

### Proliferation assays, cell cycle analysis, and apoptosis assays

The CellTiter-Glo® Luminescent Cell Viability Assay (Promega, Madison, WI) was used according to the manufacturer's instructions to estimate cell numbers. Cell cycle analyses were conducted using the fluorescein isothiocyanate BrdU Flow Kit following the manufacturer's recommendations (BD Pharmingen, San Diego, CA) as previously described [18]. Apoptotic cells were identified using Annexin-V-FLUOS Staining Kit (Roche, Mannheim, Germany) following the manufacturer's protocol. Staurosporine, 300 nM overnight was used as a positive control to induce apoptosis in TS543 cells. All flow analyses were performed 48 h post-transfection.

### Animal experiments

ICR scid (Taconic Farms Inc, Hudson, NY) 5–6 week old male mice were anesthetized with intraperitoneal injections of ketamine (0.15 mg/g) and xylazine (0.015 mg/g). An electric razor was used to shave the top of the head and the area was cleaned using alcohol and betadine. Scalps were sagittally incised to expose the skull, and a 1 mm burr hole was drilled in the skull. Injections into the subventricular zone (SVZ) were performed using a stereotactic fixation device (Kopf Instruments, Tujunga, CA, model 963). Two microliters of 1–2×10^4^ transfected TS543 cell suspension was delivered into the right hemisphere using a Hamilton syringe (Hamilton, Reno, NV catalog # 87930) over 2 minutes. Relative to Bregma the coordinates were 1.0 mm anterior, 2.0 mm lateral and at a depth of 3.0 mm from the skull. To make room for the sample, the Hamilton syringe was initially inserted 3.50 mm into the brain and then extracted 0.5 mm. The hole in the skull was closed with bone wax (Sharpoint, Vancouver, BC) and the incision was closed with a stapler clip. Mice were monitored carefully and sacrificed when they displayed symptoms of tumor development (lethargy, head tilt). T453 cells stably expressing a luciferase reporter plasmid were transfected with miR-34a or control oligonucleotides 24 hours prior to injection. Animal experiments were conducted using protocols approved by the Institutional Animal Care and Use Committees of Memorial Sloan-Kettering Cancer Center.

### Immunoblotting

Immunoblotting was performed using standard protocols with the following antibodies: MET (Cell Signaling, Danvers, MA, 25H2) 1∶1000; NOTCH1 (Cell Signaling, D6F11) 1∶1000; NOTCH2 (Cell Signaling, D76A6) 1∶1000; CDK6 (Cell Signaling, DCS83) 1∶2000; PDGFRA (Cell Signaling) 1∶1000; PI3 Kinase p85 (Millipore) 1∶14000; p-AKT, Ser473 (Cell Signaling, D9E) 1∶2000; AKT (Cell Signaling) 1∶1000; p-MDM2, Ser166 (Cell Signaling) 1∶1000; p53 (Cell Signaling, 1C12) 1∶1000, and β-Actin (Cell Signaling) 1∶10000.

### PDGFRA-3′UTR reporter assays

Two oligonucleotides encompassing the genomic sequence surrounding the two proposed miR-34a target site in the 3′ UTR of PDGFRA were synthesized (IDT DNA, Coralville, IA): 5′ TCGATATGTATATATGTATTTCTATATAGACTTGGAGAATACTGCCAAAACATTTATGACAAGCTGTATCACTGCCTTCGTTTATATTTTTTTAACTGT and 5′ GGCCACAGTTAAAAAAATATAAACGAAGGCAGTGATACAGCTTGTCATAAATGTTTTGGCAGTATTCTCCAAGTCTATATAGAAATACATATATACATA. The two oligonucleotides were annealed, and ligated into the psiCHECK22 vector (Promega, Madison, WI) using XhoI and NotI sites. TS543 cells were plated in laminin (Sigma-Aldrich, St. Louis, MO) coated wells and transfected with the psi-CHECK-2-contruct or the empty psi-CHECK2 vector using Lipofectamine LTX with Plus (Invitrogen, Grand Island, NY) according to the manufacturer's instructions. After 6 hours, cells were transferred to new wells and transfected with miRNA mimics using Hiperfect reagent (Qiagen, Valencia, CA) as described above. One day later, cells were lysed with Passive Lysis Buffer and assayed for luciferase expression using the Dual-Luciferase Reporter Assay System (Promega Madison, WI) according to the manufacturer's protocol.

### miR-34a overexpression microarray analysis

miR-34a and control oligonucleotide was transfected into TS543 cells in triplicate experiments. After 3 days, RNA was isolated and expression analyses were performed using Illumina HT-12 bead array. The microarray dataset was normalized using a variance stable normalization (VSN) procedure in the ‘lumi’ package from the Bioconductor framework [19]. Differential mRNA expression between miR-34a and control transfected samples was determined using the moderated t-statistic from the ‘limma’ Bioconductor package [20]. To systematically evaluate which 3′UTR motifs best explain mRNA down-regulation after miR-34a transfection, we used a previously published non-parametric rank-based statistic to perform an exhaustive and unbiased assessment of the correlation of mRNA 3′UTR word occurrences and the change in gene expression after miR-34a transfection [21]. In this analysis, genes were sorted by their expression change after transfection of miR-34a, and the association with down-regulation was tested for all words of length 5–7 (N = 21 504) by comparing to a null model based on dinucleotide shuffled 3′UTR sequences and random permutations of the ordered list of mRNAs (FIG. S3). The microarray data has been deposited in the public database Gene Expression Omnibus (GEO) under accession number GSE34242.

### TCGA GBM data

GBM miRNA and mRNA expression datasets (level 3) were obtained from the TCGA data portal (http://tcga-data.nci.nih.gov/tcga). The integrated dataset, having both miRNA and mRNA expression data, comprised 341 samples (TCGA sample IDs listed in Table S1). This dataset was used for testing pairwise association of miRNA and mRNA expression. Analysis relating to GBM subtypes was restricted to the 191 GBM tumor samples, which had miRNA expression data and were previously classified by the TCGA consortium [3]. This set comprised 55 proneural samples, 28 neural, 52 classical, and 56 mesenchymal samples (TCGA sample IDs listed in Table S1). For analysis relating to p53 mutation status of the tumor samples, TP53 mutation data was obtained for the same 199 samples from the cBio Cancer Genomics Portal (www.cbioportal.org/public-portal).

### MicroRNA target prediction

MicroRNAs predicted to target PDGFRA and predicted target genes of miR-34a were determined from the intersection of miRNA target predictions from miRanda (miRSVR score less than −0.1) [22] and TargetScan 5.1 (context score less than 0) [23].

### Other statistical analyses

Pairwise association of miRNA and mRNA expression was evaluated by the non-parametric Spearman correlation coefficient and the associated p-value was determined by the R statistical software framework (http://www.r-project.org/). Differential miRNA expression between two groups of samples was determined using the non-parametric Wilcoxon rank-sum test (one-tailed test) in the R statistical software framework.

## Supporting Information

Figure S1 **Validation of miR-34a expression in GBM cells.** miR-34a expression measured by TaqMan 24 hours following transfection of 100 nM miR-34a or negative control microRNA to TS543 and TS600 cells.
(TIF)

Figure S2 **In proneural TS543 cells, miR-34a has minimal repressive effects on other known GBM targets.** TS543 cells were transfected with either miR-34a or control oligonucleotides for 24 hours, and MET, CDK6, and NOTCH2 protein levels were measured by immunoblotting.
(TIF)

Figure S3 **3′UTR motif analysis after miR-34a overexpression.** We systematically analyzed all words of length 5–7 (N = 21 504) for overrepresentation in down-regulated mRNAs after miR-34a transfection. Consistent with many previous studies of miRNA overexpression, the word most correlated with down-regulation was the seed site complementary to mature miR-34a bases 2–7. The figure shows the top-10 words most correlated with down-regulation. Word correlation Z-score, rank and estimated false discovery rate are indicated in columns to the right of each word. Capital letters highlight the words matching the seed region (bases 2–8) of the miRNA. Five words (shown in grey) did not align with the miR-34a sequence.
(TIF)

Table S1 TCGA sample IDs of samples used in this study.
(XLSX)
