# Supplementary figures and images for: miR-34a Repression in Proneural Malignant Gliomas Upregulates Expression of Its Target PDGFRA and Promotes Tumorigenesis
Source: PLoS One. 2012 Mar 27;7(3):e33844. doi: 10.1371/journal.pone.0033844 (PMC3313940; doi:10.1371/journal.pone.0033844)

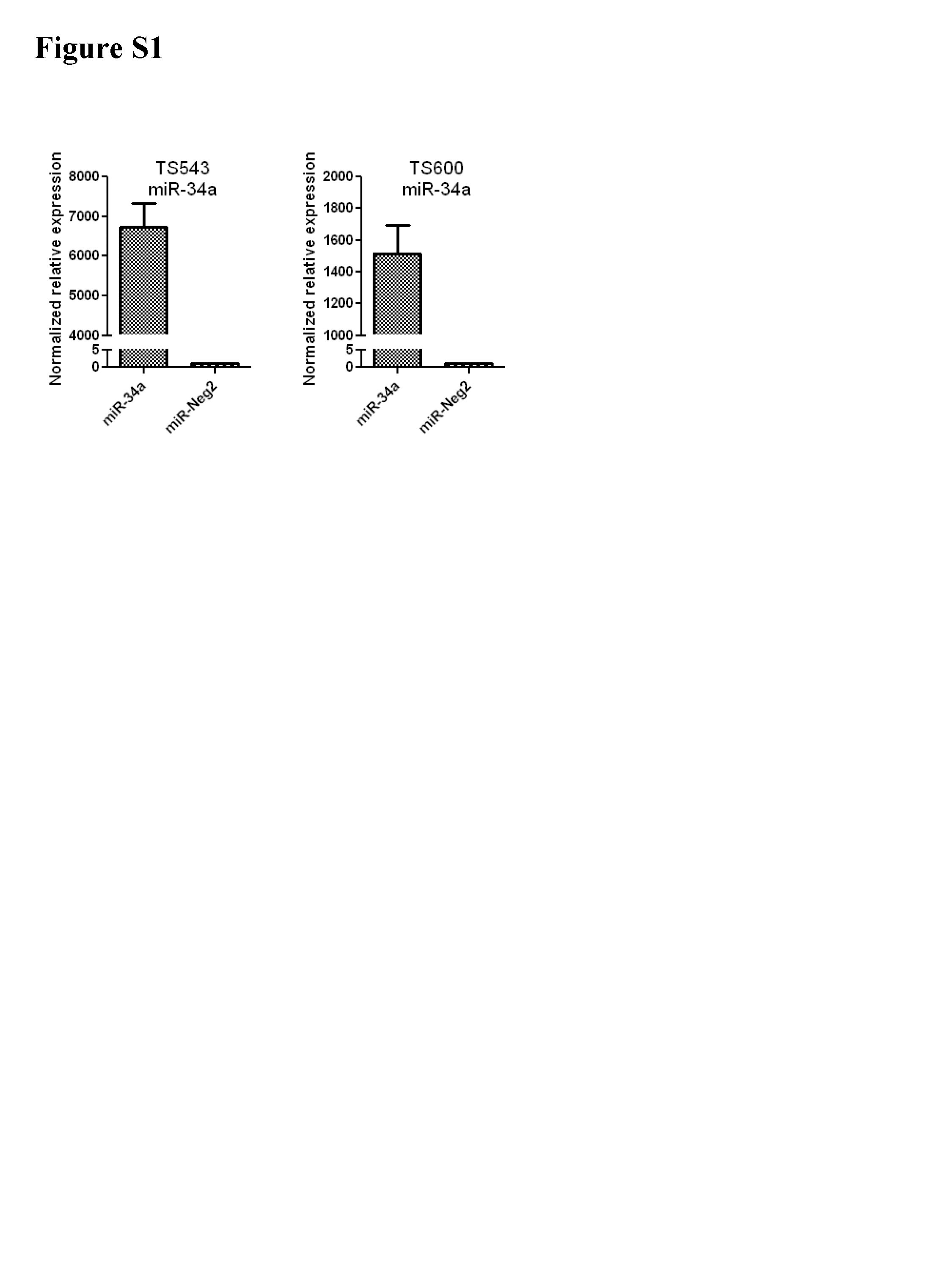

Supplement: Figure S1 — Validation of miR-34a expression in GBM cells. miR-34a expression measured by TaqMan 24 hours following transfection of 100 nM miR-34a or negative control microRNA to TS543 and TS600 cells. (TIF) [file pone.0033844.s001.tif]

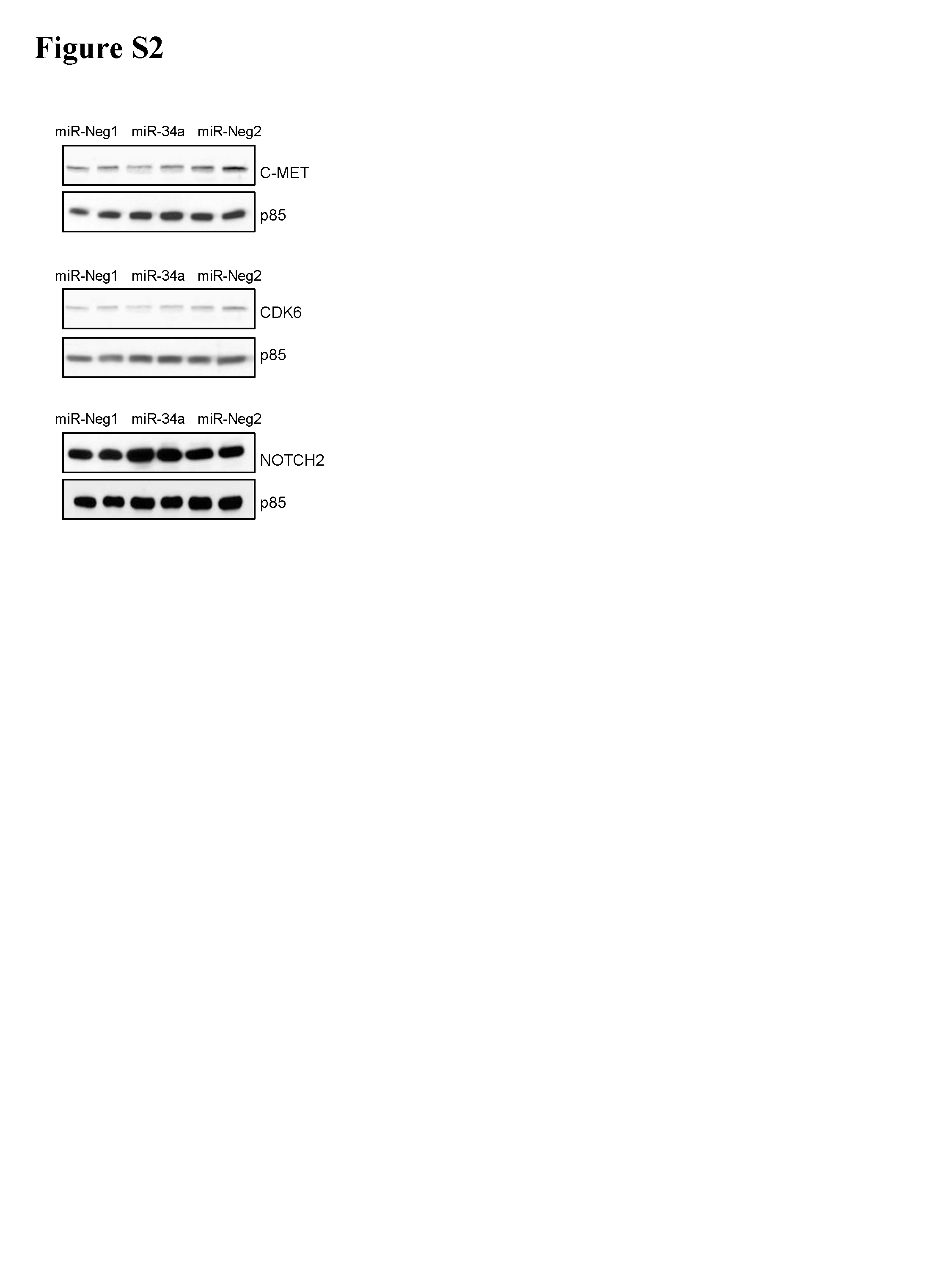

Supplement: Figure S2 — In proneural TS543 cells, miR-34a has minimal repressive effects on other known GBM targets. TS543 cells were transfected with either miR-34a or control oligonucleotides for 24 hours, and MET, CDK6, and NOTCH2 protein levels were measured by immunoblotting. (TIF) [file pone.0033844.s002.tif]

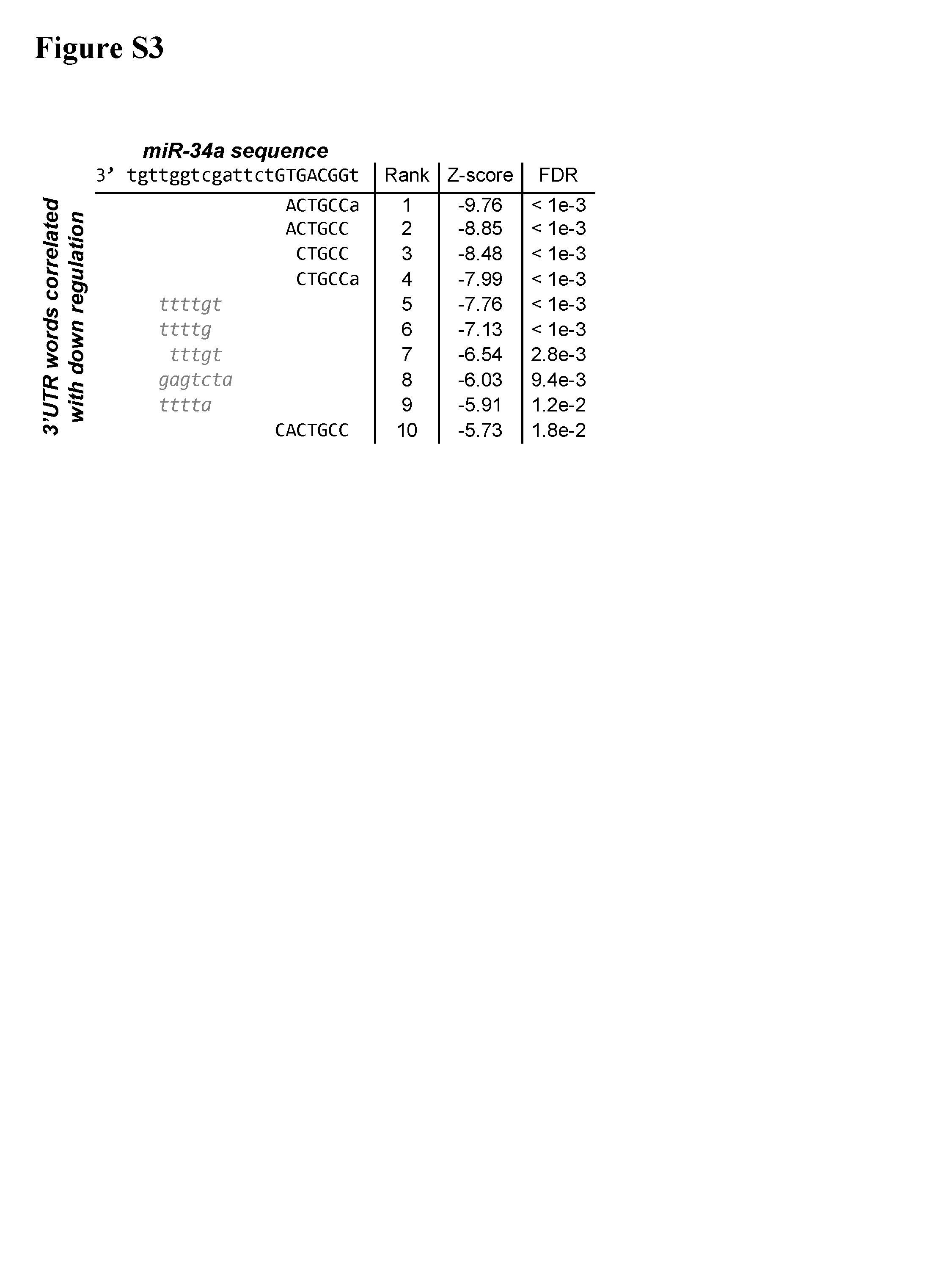

Supplement: Figure S3 — 3′UTR motif analysis after miR-34a overexpression. We systematically analyzed all words of length 5–7 (N = 21 504) for overrepresentation in down-regulated mRNAs after miR-34a transfection. Consistent with many previous studies of miRNA overexpression, the word most correlated with down-regulation was the seed site complementary to mature miR-34a bases 2–7. The figure shows the top-10 words most correlated with down-regulation. Word correlation Z-score, rank and estimated false discovery rate are indicated in columns to the right of each word. Capital letters highlight the words matching the seed region (bases 2–8) of the miRNA. Five words (shown in grey) did not align with the miR-34a sequence. (TIF) [file pone.0033844.s003.tif]
